# Pathogenic Mechanisms Underlying Cirrhotic Cardiomyopathy

**DOI:** 10.3389/fnetp.2022.849253

**Published:** 2022-04-19

**Authors:** Hongqun Liu, Henry H. Nguyen, Ki Tae Yoon, Samuel S. Lee

**Affiliations:** ^1^ Liver Unit, University of Calgary Cumming School of Medicine, Calgary, AB, Canada; ^2^ Liver Center, Pusan National University Yangsan Hospital, Yangsan, South Korea

**Keywords:** cirrhosis, cardiac, ventricular, systolic, diastolic, heart failure, pathophysiology

## Abstract

Cardiac dysfunction associated with cirrhosis in the absence of preexisting heart disease is a condition known as cirrhotic cardiomyopathy (CCM). Cardiac abnormalities consist of enlargement of cardiac chambers, attenuated systolic and diastolic contractile responses to stress stimuli, and repolarization changes. CCM may contribute to cardiovascular morbidity and mortality after liver transplantation and other major surgeries, and also to the pathogenesis of hepatorenal syndrome. The underlying mechanisms of CCM are poorly understood and as such medical therapy is an area of unmet medical need. The present review focuses on the pathogenic mechanisms responsible for development of CCM. The two major concurrent mechanistic pathways are the inflammatory phenotype due to portal hypertension, and protein/lipid synthetic/metabolic defects due to cirrhosis and liver insufficiency. The inflammatory phenotype arises from intestinal congestion due to portal hypertension, resulting in bacteria/endotoxin translocation into the systemic circulation. The cytokine storm associated with inflammation, particularly TNFα acting via NFκB depresses cardiac function. They also stimulate two evanescent gases, nitric oxide and carbon monoxide which produce cardiodepression by cGMP. Inflammation also stimulates the endocannabinoid CB-1 pathway. These systems inhibit the stimulatory beta-adrenergic contractile pathway. The liver insufficiency of cirrhosis is associated with defective synthesis or metabolism of several substances including proteins and lipids/lipoproteins. The protein defects including titin and collagen contribute to diastolic dysfunction. Other protein abnormalities such as a switch of myosin heavy chain isoforms result in systolic dysfunction. Lipid biochemical changes at the cardiac sarcolemmal plasma membrane result in increased cholesterol:phospholipid ratio and decreased membrane fluidity. Final common pathway changes involve abnormal cardiomyocyte intracellular ion kinetics, particularly calcium. In conclusion, cirrhotic cardiomyopathy is caused by two pathways of cellular and molecular dysfunction/damage due to hepatic insufficiency and portal hypertension.

## Introduction

Cirrhosis is invariably associated with two major abnormalities, although the severity of each abnormality can vary, usually correlated to the stage of cirrhosis. One is liver failure, i.e., hepatocellular insufficiency with defective synthesis or metabolism of several moieties including proteins and lipids. The other is portal hypertension, resulting in a congested gut (Table 1).

**TABLE 1:** Main messages.

1. The new diagnostic criteria (2020 CCC system) have replaced the older criteria. Montreal (2005).
2. Cirrhotic cardiomyopathy (CCM) affects the prognosis and outcomes after surgery/procedures such as liver transplantation and transjugular intrahepatic portosystemic shunt.
3. Liver failure increases vasoactive factors and changes the components of important proteins/lipids in cardiomyocytes.
4. Portal hypertension results in intestinal vascular congestion, bacterial translocation with endotoxemia, and ‘cytokine storm’ of the inflammatory phenotype.
5. Therefore the alterations associated with liver failure and portal hypertension play key pathogenic roles in CCM.

Likely mostly related to the congested gut, perturbations in the gut microbiota have been documented in cirrhosis. Various studies have highlighted alterations in the gut microbiome of cirrhotic patients yielding overall lower microbial gene richness, and increase in the prevalence of oral commensals (Bajaj et al., 2014), The exact mechanism linking the luminal gut microbiota to downstream liver health outcomes is not fully elucidated in the patient setting. Current studies have implicated the microbiome in cirrhosis highlighting benefit in fecal microbial transplant in early phase 1 studies evaluating its effect on hepatic encephalopathy (Bajaj et al., 2021). Clinical studies have also highlighted an association between gut microbiota signature as a predictor of liver fibrosis in patients with non-alcoholic fatty liver disease (Oh et al., 2020). Although promising, the current existing studies have yet to delineate the mechanisms by which the host luminal microbiota can affect downstream liver and systemic health. Animal models have highlighted potential pathways which may also be occurring in the clinical setting. Variables including altered gut barrier function and altered luminal microbially derived metabolites (short chain fatty acid, pathogen associated molecular patterns, cytokines, bile acids, etc) acting on parenchymal and immune cells in distant organs to affect disease outcomes have been described (Albhaisi et al., 2020; Lang and Schnabl, 2020).

The role of the microbiota in mediating cardiac disease outcomes has also been evaluated with studies finding perturbations in microbial content and metabolites potentially able to mediate cardiac dysfunction and thus overall cardiovascular disease outcomes (Beale et al., 2021). Taken together, the intra-abdominal changes associated with portal hypertension including mesenteric ischemia, alterations in gut barrier function with increase in intestinal permeability, luminal bacterial overgrowth and ultimately translocation of bacteria and or bacteria derived products that result in endotoxemia, can potentially lead to an elevation of systemic pro-inflammatory cytokines including, but not limited, to tumor necrosis factor alpha (TNFα), interleukin 1β (IL-1β) and interleukin-6 (IL-6) (Liu et al., 2000; Yu et al., 2021), that can then mediate downstream cardiac dysfunction. Currently there is a paucity of both experimental and clinical data evaluating the role of the microbiota in cirrhosis associated cardiomyopathy.

In cirrhosis, inflammatory cytokines TNFα, IL-1β (Liu et al., 2000) and IL-6 (Yu et al., 2021) are increased in cardiac tissue in cirrhotic animal models. The cardiac inflammation mediates damage to the heart which include apoptosis and hypertrophy of the cardiomyocytes, and increased rigidity of the cardiac membrane (Ma et al., 1997). Furthermore, the increased systemic inflammation resulting from the altered circulation with portal hypertension causes peripheral vascular dilatation which decreases systemic vascular resistance and overall mean arterial blood pressure. Compensatory activation of the sympathetic nervous system and its effect on the β1-adrenergic receptors in the cirrhotic heart may also further damage cardiac tissue (Yoon et al., 2021). Additional factors, including endocannabinoids have also been implicated to play important roles in the pathogenesis of cirrhotic cardiomyopathy (CCM), by way of its direct effect and signaling through G-protein-coupled receptors.

Hepatic insufficiency is associated with myriad defects in metabolism or synthesis of moieties including proteins and lipids. In the heart, the giant protein titin and collagen isoforms are involved in regulation of intrinsic compliance/stiffness and thus are crucial in diastolic function. Myosin heavy chain (MHC) is the so-called ‘molecular motor’ of contractility. Lipids and lipoprotein synthesis/metabolism are deranged in cirrhosis and thus lead to abnormal lipid content and function of cardiac sarcolemmal plasma membranes. The bile acid profile of cirrhosis is significantly altered and these contribute to pathogenesis of CCM. Recent preliminary evidence has even suggested that the lectin family, specifically galectin-3, may play a role in pathogenesis of CCM.

Thus changes in both the congested gut and in liver failure by several distinct mechanistic pathways result in the manifestations of CCM, mediated by the final common pathways of abnormal transmembrane and intracellular ion function, particularly the key ion involved in contraction, calcium. This review summarizes the definition and diagnostic criteria of CCM, and its clinical relevance, but will focus mainly on clarifying in detail the underlying mechanistic pathways responsible for this condition.

## Disruption of Cardiovascular-Hepatic Network Connectivity/Crosstalk

Befitting the theme of this special issue, it is thus very clear that the heart-liver network connectivity is significantly disrupted at several levels due to chronic liver disease. That of course is only half the problem: in acute or chronic heart failure, the liver networks are also disrupted due to hypoxic hepatitis or congestive hepatopathy. That issue is beyond the scope of this issue and thus not covered here. Herein we confine our discussions to the heart when the liver fails, not the converse.

The many changes/disruptions of substances made or metabolized by the liver such as proteins, lipids, lectins, bile salts, and hormones due to hepatic insufficiency, contribute significantly to dysfunction of cardiovascular networks in cirrhosis as we will clarify in the following pages. Just as importantly, cirrhosis with dense liver fibrosis and abnormal central neural cardiovascular-regulatory dysregulation (Song et al., 2002; Liu et al., 2008) producing hyperdynamic circulation and portal hypertension with gut congestion is the major cause of the inflammatory phenotype which disrupts the cardiovascular network. Therefore, there is intricate and complex crosstalk/connectivity between liver and cardiovascular networks which is severely disrupted by cirrhosis and liver failure.

## Definition and Diagnostic Criteria

Cirrhotic cardiomyopathy is defined as subnormal cardiac function in the absence of prior heart disease (Chahal et al., 2021). The heart at resting status manifests no cardiac dysfunction due to peripheral vasodilation in cirrhotic patients and the cardiac output is normal or increased (Kaur and Premkumar, 2022). When challenged, the cardiac function is overt. Based on these ideas, the initial diagnostic criteria were formulated at the 2005 World Congress of Gastroenterology in Montreal (Liu et al., 2017).

In brief, the proposed criteria included measurements of systolic and diastolic dysfunction at rest and under stress challenges, myocardial chamber enlargement, electrophysiological abnormalities and biomarkers such as Troponin and brain natriuretic peptide (BNP).

Since the creation of those criteria, advances in imaging techniques have been developed. These advanced imaging techniques allow detection of cardiac abnormalities, such as diastolic and systolic indices, at rest (Izzy et al., 2020). Furthermore, knowledge of heart failure has also significantly advanced, particularly regarding the detailed characterization of heart failure into two forms, one with preserved ejection fraction (now labelled heart failure with preserved ejection fraction, HFpEF) and the other with reduced ejection fraction (HFrEF). Cirrhotic cardiomyopathy belongs to the HFpEF subtype. These diagnostic and conceptual advances have essentially rendered the initial 2005 CCM criteria obsolete (Table 2; Liu and Lee, 2021).

**TABLE 2:** Diagnostic criteria are as follows (Liu et al., 2017).

1. Abnormal systolic contractile responses to stress.
2. Diastolic dysfunction at rest.
3. Absence of clinically significant cardiopulmonary disease.
Systolic dysfunction (at least 1 of the following):
1. Blunted increase in cardiac output with exercise, volume challenge, or pharmacological stimuli.
2. Resting left ventricular ejection fraction (LVEF) <55%.
Diastolic dysfunction (at least 1 of the following):
1. The ratio of peak early (E wave) and atrial (A wave) flow velocities (E/A; early/late diastolic filling velocities) ratio (age corrected) < 1.0.
2. Prolonged mitral deceleration time (DT; >200 ms).
3. Prolonged isovolumic relaxation time (>80 ms).
Supportive criteria:
1. Electrophysiological abnormalities including the following:
a. Abnormal chronotropic response to stress.
b. Electromechanical uncoupling/dysynchrony.
c. Prolonged QTc interval.
2. Heart chamber alterations: enlarged left atrium (LA) and increased left ventricular wall thickness.
3. Increased pro–brain-type natriuretic peptide (BNP) and BNP.
4. Increased troponin I.

In 2020, the Cirrhotic Cardiomyopathy Consortium (CCC), which is composed of a multidisciplinary expert group, redefined the diagnostic criteria of cirrhotic cardiomyopathy (Izzy et al., 2020). These criteria, unlike the 2005 proposal, are based solely on the cardiac abnormalities at rest, which are detected by advanced techniques such as echocardiographic strain analysis and tissue Doppler imaging. The new proposed criteria are listed in Table 3.

**TABLE 3:** Diagnostic criteria proposed by Cirrhotic Cardiomyopathy Consortium

Systolic Dysfunction	Advanced Diastolic Dysfunction	Areas for Future Research
Any of the following	≥3 of the following	• Abnormal chronotropic or inotropic response§
• LV ejection fraction ≤50%	• Septal e′ velocity <7 cm/s	• Electrocardiographic changes
• Absolute* GLS <18%	• E/e′ ratio ≥15	• Electromechanical uncoupling
	• LAVI >34 ml/m2	• Myocardial mass change
	• TR velocity >2.8 m/s	• Serum biomarkers
		• Chamber enlargement
		• CMRI

GLS, global longitudinal strain; e′, early diastolic mitral annular velocity; E/e′, ratio of mitral peak velocity of early filling to early diastolic mitral annular velocity; CMRI, cardiac magnetic resonance imaging.

Some studies have used the new criteria to calculate the prevalence of CCM. Unfortunately, all the studies used a global longitudinal strain (GLS) score <18% or >22% as abnormal criteria (Cesari et al., 2021; Razpotnik et al., 2021; Kaur and Premkumar, 2022). This is because the original publication (Izzy et al., 2020) contained an error in the GLS criteria: it should have only been <18%, and not also >22%. The error was undetected for several months until a corrigendum was electronically published later in 2020, but by then it had caused some confusion and ‘damage’. With the error of GLS ≥22% included in the study of Cesari et al., they demonstrated that the prevalence of CCM is 29% in cirrhotic patients. However, this study lumped the patients with GLS <18% and GLS ≥22% together. Therefore, we cannot differentiate these two groups and thereby determine the correct prevalence of CCM in their study. Interestingly, these investigators (Cesari et al., 2021) have also raised questions about the usefulness of LVEF <50% as a criterion because no patient in their study had LVEF <50%. They suggested modifying the criteria by removing the LVEF and adding a stress test to assess the cardiac contractile reserve.

Although the new criteria have been published online for more than 2 years, to date, only Razpotnik and colleagues (Razpotnik et al., 2021) have systematically compared the Montreal and the CCC criteria. They found that overall prevalence of CCM was similar for the Montreal (67.2%) and CCC (55.7%) criteria. However, if the patients with GLS ≥22% are excluded, the prevalence of CCM by the new CCC criteria should be only 19.7% (Liu and Lee, 2021).

The 2020 CCC criteria have not yet been firmly demonstrated to have prognostic or other utility, and some questions remain. For example, all contractile indices are measured under resting conditions and not under any stress challenge. However, at present, we believe that this system represents the best way forward for clinical and prognostic studies and trials.

## Clinical Relevance

CCM, although subclinical in resting status, is significant because when the cardiovascular system is challenged, such as by liver transplantation, transjugular intrahepatic portosystemic shunt (TIPS), drugs, and exercise (Yoon et al., 2020), cardiac dysfunction can become overt. Liver transplantation challenges the cardiovascular system. Intravenous fluids augments preload, an increased systemic vascular resistance elevates afterload, and therefore, liver transplantation significantly increases the cardiac workload which aggravates the preexisting CCM. Cardiac events such as arrhythmias, angina, and heart failure decrease the rates of patient and graft survival (Liu et al., 2021). Indeed, cardiovascular complications are the third leading cause of death, after infections and rejection, in liver transplant recipients. It is estimated that 7–21% of deaths after liver transplantation are due to cardiovascular complications (Liu et al., 2017).

CCM is associated with mortality in cirrhosis. Premkumar et al. found that mortality rates were correlated with the grades of left ventricular diastolic dysfunction (LVDD grade 1, 10.5%, 2, 22.5% and 3, 40%) within 2 years (Premkumar et al., 2019). They also revealed that the severity of LVDD also plays an important role in the development of acute kidney injury (OR 6.273, *p* < 0.05) and hepatic encephalopathy (OR 5.6, *p* < 0.05).

The study by Ruiz-del-Arbol et al. (2005) demonstrated that in patients with resolving spontaneous bacterial peritonitis, lower cardiac output is significantly correlated with the development of hepatorenal syndrome (HRS). Krag et al. (2010) also showed that the number of patients who developed HRS type 1 within 3 months was higher in patients with low cardiac index than in those with high cardiac index (43 vs 5%, *p* = 0.04). These studies suggest that inadequate systolic contractile response to a significant cardiovascular challenge posed by infection or the peripheral vasodilatation of end stage cirrhosis, with reduced renal perfusion, contributes to the pathogenesis of acute kidney injury and hepatorenal syndrome.

An important recent topic in cirrhosis is the so-called ‘frailty syndrome’ with severe muscle wasting, and general debility with impaired mobility (Tandon et al., 2021). Underlying this is severe sarcopenia. No studies have yet examined this, but we wonder whether the reduced skeletal muscle perfusion caused by cirrhotic cardiomyopathy may play a role in its pathogenesis? It is also possible and perhaps even probable, that many of the mechanisms we describe below in the cardiac myocyte may also apply to skeletal muscle myocytes.

## Pathogenic Mechanisms

**FIGURE 1:**
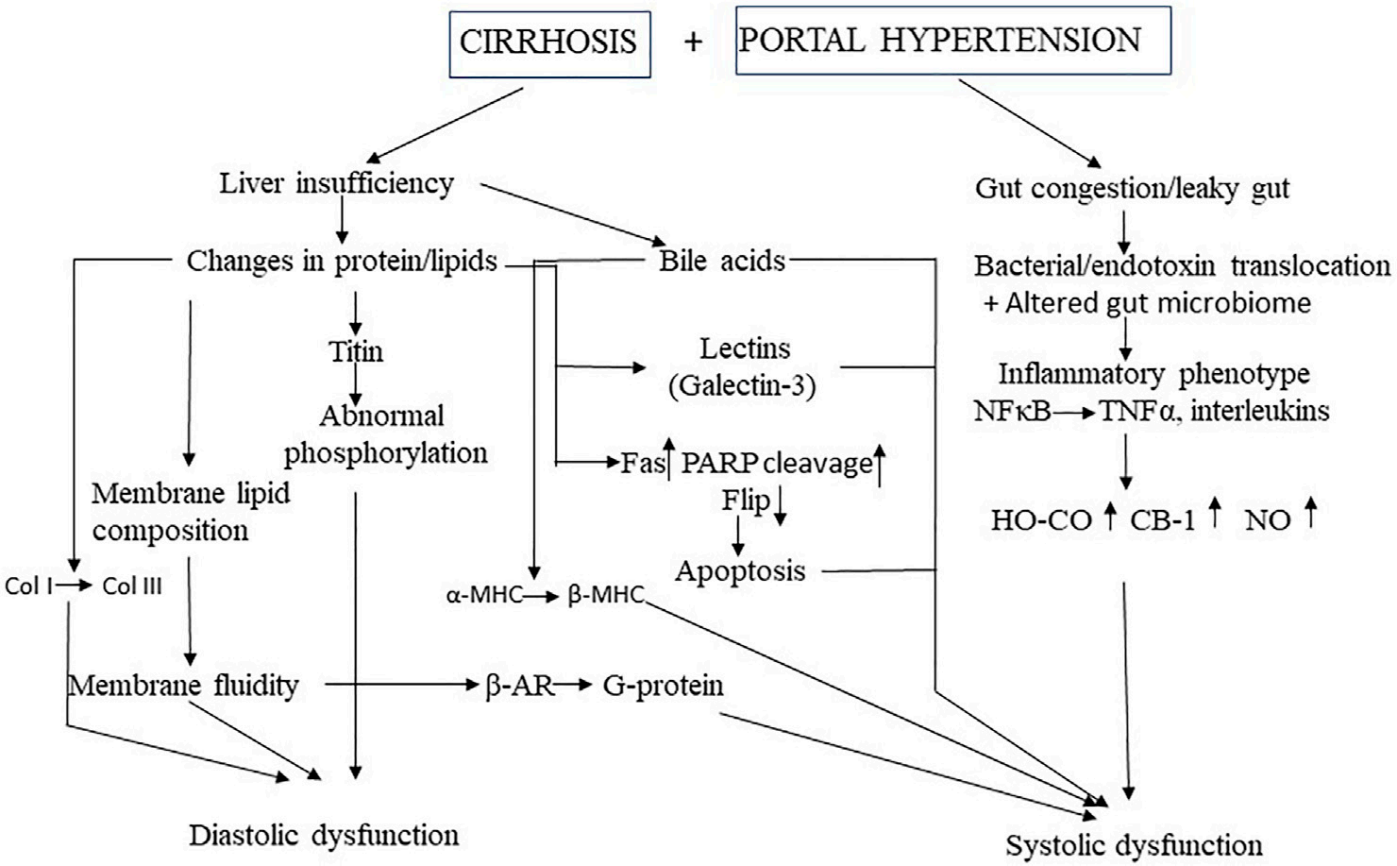
Mechanistic pathways of cirrhotic cardiomyopathy MHC, myosin heavy chain: Col, collagen, TNF, tumor necrosis factor, HO, heme oxygenase, Co, carbon mononide; CB, cannabinoid; NO, nitric oxide, β-AR, β-adrenergic receptor, PARP, poly(ADP-ribose) polymerase; FLIP, Fas-associated death domain-like interleukin 1β-converting enzyme inhibitory protein.

There are two concurrent pathways in the pathogenesis of CCM: liver failure and portal hypertension (Figure 1). The former increases vasoactive factors such as bile acids in the circulation, changes the membrane components of cardiomyocytes and important proteins such as the contractile filaments inside cardiomyocytes. The latter causes intestinal vascular congestion, bacterial translocation with endotoxemia, and the resultant “cytokine storm” of the inflammatory phenotype. At present, the relative importance of the many sub-mechanisms described below remains unclear, ie., it is unknown if some are more dominant than others. It should also be noted that many studies of a single system or pathway, for example, nitric oxide inhibition, completely reverses the cardiac dysfunction, but this is explained by the experimental protocol with manipulation of a single system and the others held constant, in an *in vitro* preparation, in the vast majority of studies. In the few *in vivo* studies, the reasons for a complete reversal with a single pathway inhibited remain unclear, but we speculate that this may be due in part to the protocol with acute blockade or inhibition of a single system, thus not permitting sufficient time for other compensatory or additive mechanisms to exert any effects.

## Liver Failure

### Bile Acids

Serum concentration of bile acids have been characterized to be elevated in cirrhotic patients (Neale et al., 1971; Salhab et al., 2021). Bile acids have complex structural and biochemical properties and therefore, have pleiotropic functions that extends beyond its current use as a biomarker of liver dysfunction (Khurana et al., 2011). Bile acids have been shown to decrease cardiac contractility in basal and cardiac beta-adrenoceptor-stimulation (Zavecz and Battarbee, 2010). The inhibitory mechanisms of bile acids on cardiac contractility are thought to occur via different mechanisms which include:

1) A switch from a rapid α-myosin heavy chain (MHC) to β-MHC isoform. Desai and colleagues demonstrated that high-dose bile acids in a murine model of cholestasis facilitate the switch of MHC from α-MHC to β-MHC isoform (Desai et al., 2010; Desai et al., 2017).
2) Disruption of calcium homeostasis: bile acids can cause abnormal calcium dynamics in cardiomyocytes (Miragoli et al., 2011). Specifically, Gorelik et al. (2002). found that taurocholate, the product of conjugation of cholic acid with taurine, significantly reduced calcium transients. They further observed that taurocholate affects calcium release from the sarcoplasmic reticulum which parallels the inhibition of cardiac contractile function. There was a dose response in this study with significant inhibition with higher concentrations. The inhibitory effects on cardiac contraction were reversible following the removal of taurocholate. Administration of tauroursodeoxycholic acid (TUDCA), a chemical chaperone and bile salt used to treat hepatobiliary disorders, improved cardiomyocyte contractile and intracellular Ca^2+^ anomalies (Turdi et al., 2013).
3) Effect on papillary muscles: Zavecz and Battarbee (2010) tested the effects of cholic acid, a lipophilic bile acid, on Ca^2+^ influx in an otherwise normal tissue. They found that under the environment of 100 µM cholic acid, the amount of tension of right ventricular muscle fiber bundles that was developed at different Ca^2+^ concentrations was significantly less than that of control. The behavior of papillary muscles under the effect of cholic acid at 100 µM mimics that from portal vein stenosis and CCl4-induced cirrhotic rats (Zavecz and Battarbee, 2010).
4) Effect on cardiac contractility through shared molecular structure: bile acids has also been demonstrated to inhibit cardiac contractility via M2-muscarinic receptor. Raufman et al. (2002) observed that the molecular surface structures of acetylcholine and the bile acids are similar. The inhibitory effect of bile acids on cardiac contractility is via the stimulation of bile acids on M2-muscarinic receptor which inhibits intracellular cAMP production (Sheikh Abdul Kadir et al., 2010). Pharmacological inhibition and siRNA-knockdown of the M2-muscarinic receptor abolishes the negative effect of bile acid on cardiac contraction (Sheikh Abdul Kadir et al., 2010).
5) Mediating a metabolic switch in cardiomyocytes. In the normal heart, 60% of the energy are from the oxidation of fatty acids, with 40% from carbohydrates (Taha and Lopaschuk, 2007). Bile acids have been shown to mediate the alteration of energy substrate from fatty acid to glucose ultimately driving contractile dysfunction. The switch from fatty acid to glucose metabolism has been shown to occur in various cardiomyopathies, such as dilated cardiomyopathy (Dávila-Román et al., 2002) and hypertrophic cardiomyopathy (Gehmlich et al., 2015). Since alterations in myocardial substrate metabolism are implicated in the pathogenesis of contractile dysfunction and heart failure (HF) (Dávila-Román et al., 2002) and bile acid has been implicated in the alterations of energy substrate from fatty acid to glucose (Desai et al., 2017), the inhibition of cardiac contractility of bile acid may be partially due to the energy **s**ubstrate change in cirrhotic cardiomyopathy.

## Cardiac Plasma Membrane Physical Properties

All cardiac function-related receptors and ion channels, such as adrenergic receptors, muscarinic receptors, cannabinoid receptors, L-type calcium channels, and Na⁺/K⁺-ATPase etc. are embedded into the plasma membrane of cardiomyocytes, which consists of a phospholipid bilayer. Any changes of the biochemical components affects the fluidity and the functions of proteins implanted in the plasma membrane and their downstream signaling pathways. Our group has shown that the cholesterol-to-phospholipid ratio is increased in cardiac membranes from cirrhotic rats, which results in the increase in structural rigidity of the plasma membrane. The alteration of cardiac membrane impacts the function of β-adrenergic receptors and furthermore, decreases cAMP generation. Using 2-(2-methoxyethoxy)ethyl 8-(cis-2-n-octylcyclopropyl) octanoate (A2C) to restore the fluidity of cardiac membranes of the cirrhotic rats to that of control values, cAMP production stimulated with β-adrenergic receptor against, isoproterenol, is significantly increased. This observation demonstrated that the blunted cardiac contractility in cirrhotic rats is in part due to the increased membrane rigidity which results in the decrease of β-adrenergic receptor function (Ma et al., 1994; Ma et al., 1997).

## Myofilaments

Titin abnormalities are associated with dilated cardiomyopathy (Kellermayer et al., 2019). Titin has three isoforms, N2A, N2B and N2BA. N2B and N2BA are isoforms expressed within adult cardiomyocytes. N2B is a comparatively shorter protein with less elasticity and more passive stiffness compared to the N2BA (Tharp et al., 2019). The increased ratio of N2BA:N2B titin isoforms has been suggested to play an important role in patients with dilated cardiomyopathy (Nagueh et al., 2004). Utilizing the bile duct ligation model in rats, our group however did not observe a change in the ratio of N2BA:N2B titin isoforms. Instead we demonstrated that decrease in the levels of cardiac titin modulator, protein kinase A, plays a pathogenic role in cirrhotic cardiomyopathy. Furthermore, we also observed that the stiffer collagen I was significantly increased and the more compliant collagen III was significantly decreased in the cirrhotic rats compared with sham controls, which may explain the presence of diastolic dysfunction in cirrhotic cardiomyopathy (Glenn et al., 2011).

Another myofilament that plays an essential role in cardiac contraction is myosin heavy chain (MHC), the ‘molecular motor’ of the heart (Miyata et al., 2000). There are two isoforms of MHC, alpha and beta that exist in the mammalian ventricular myocardium. Both types of MHCs are coexpressed together in control animals. The ventricular β-MHC is the dominant isoform in the late fetal life in the rats. α-MHC increases with age and almost completely replaces β-MHC in adult rats. However, many stimuli, including thyroid depletion, alcohol, cardiomyopathy, and pressure overload, can mediate a switch in the MHC isoform from α-MHC to β-MHC in adult (Miyata et al., 2000; Shirpoor et al., 2017). MHC isoform changes has been illustrated by others to play a major role in the determination of cardiac contractility (Miyata et al., 2000). Their relative expressions has been shown to determine the contractile velocity of cardiac muscle (Nakao et al., 1997).

Nakao et al. compared the MHCα mRNA expressions in the left ventricular free walls from normal heart and that from chronic end-stage heart failure, and found that α-MHC mRNA expression is significantly decreased in the setting of cardiac dysfunction (Nakao et al., 1997). Huang and coworkers also revealed that the ratio of MHC-β to MHC-α mRNA increased 5-fold in dietary hypercholesterolemia induced “cholesterol cardiomyopathy" (Huang et al., 2004). Miyata et al. demonstrated that the switch of MHC from alpha type to beta type in heart failure also occurs at the protein level; corroborating the observations of changes in the transcription levels described by Nakao et al. (Miyata et al., 2000). Our recent study found that the α-and β-isoforms of myosin heavy chain (MHC) are significantly altered in a rat model of cirrhotic cardiomyopathy (Honar et al., 2020). Specifically, the stronger, faster contracting α-subtype was replaced by the weaker, slower-contracting β-MHC. Since the switch from α-MHC to β-MHC plays an important role in cardiac dysfunction (Huang et al., 2001), this switch may also play a role in cirrhotic cardiomyopathy.

## Portal Hypertension and the Inflammatory Phenotype

### Monocyte/Macrophage Infiltration

Monocytes/macrophages innate immune cells have been shown to infiltrate hypertrophic hearts (Abbadi et al., 2018) which can cause systolic dysfunction. Local inflammation within the myocardium is mediated by macrophage derived miR-155 which promotes inflammation, hypertrophy, and failure to respond to pressure overload (Heymans et al., 2013). In the setting of cirrhosis, we found (Gaskari et al., 2015) increased monocyte recruitment in the myocardium and decreased myocardial contractility. In this study, isolated monocytes from heart tissue in cirrhotic rats inhibited cardiomyocyte contractility to a greater degree than those from sham control *in vitro*. Hemorrhage further increased the monocyte infiltration in cirrhotic heart. Furthermore, preventing monocyte recruitment with gadolinium chloride significantly improved cardiac contractility in cirrhotic rats, and restored cardiovascular response to blood loss (Gaskari et al., 2015).

The role of monocytes and macrophages in animal models of cardiac dysfunction, is thought to be in part mediated by an increase in the production of pro-inflammatory cytokines such as TNFα and IL-6 (Huo et al., 2021; Nemec Svete et al., 2021). The inhibition of these pro-inflammatory cytokines likewise has been demonstrated to alleviate myocardial inflammation, cardiac remodeling, and contractile dysfunction (Chang et al., 2021; Huo et al., 2021). Our group has previously demonstrated that pro-inflammatory cytokines such as TNFα and IL1β are significantly increased in cirrhotic rat hearts (Liu et al., 2000; Yang et al., 2010). Specifically, we observed TNFα depressing cardiac contractility with cirrhosis, and blockage of this cytokine using anti-TNFα antibody significantly improved systolic and relaxation velocities in cardiomyocytes from cirrhotic mice (Yang et al., 2010).

### Oxidative Stress

Oxidative stress is significantly increased in heart failure in both canine and murine models (Huo et al., 2021; Nemec Svete et al., 2021). Using abdominal aortic constriction model in rats, Chi et al. found that the oxidative stress was increased and associated with decreased left ventricular fractional shortening at 4 weeks after surgery. Using the readily available and well characterized antioxidant N-acetylcysteine (NAC), treatment in cirrhotic animals significantly attenuated the decreased left ventricular fractional shortening (Chi et al., 2021). Our group has demonstrated that oxidative modified proteins are significantly increased, while nuclear factor (erythroid-derived 2)-like 2 (Nrf2), a protein that regulates the expression of antioxidant proteins, is significantly decreased in cardiac tissue from cirrhotic rats (Liu et al., 2012).

Erythropoietin has also been characterized to have antioxidant properties in addition to its role in red blood cell production. Studies have found that erythropoietin administration in cirrhotic rats can significantly decreases the levels of oxidative protein and increases Nrf2 in cardiac tissue. This has been shown to ultimately improve cardiac function in the setting of cirrhosis (Liu et al., 2012).

### Apoptosis

Apoptosis plays an important role in myocardial remodeling and heart failure. Reducing cardiomyocyte apoptosis has been shown to attenuate chronic heart failure (Nastro et al., 2021). Similarly, our group has previously demonstrated that apoptosis plays a crucial role in CCM (Nam et al., 2014). Specifically, we found that poly(ADP-ribose) polymerase (PARP) cleavage, a marker of apoptosis, and Fas protein expression, an index of extrinsic apoptotic pathways, are significantly increased in the cirrhotic rat heart. Furthermore, B-cell lymphoma 2 (Bcl-2), an anti-apoptosis parameter, is also significantly increased. The Bcl-2/Bax ratio is increased in cardiac tissue from cirrhotic mice compared with sham controls suggesting the external apoptotic pathway is predominant in the overall the pro- and anti-apoptotic balance. Disruption of apoptosis via anti-FasL monoclonal antibody injection in cirrhotic mice improves overall systolic and diastolic dysfunction in isolated cardiomyocytes compared to control mice. Overall, our findings suggest that a pro-apoptotic balance exists in cardiac tissue from cirrhotic mice that is in part mediated by activation of the extrinsic apoptotic pathway. Abrogation of apoptosis improved cardiac contractility.

### Nitric Oxide

The role of NO in cardiac contraction is concentration dependent. At low concentration, NO inhibits phosphodiesterase III, preventing the hydrolysis of cAMP. The increased cAMP subsequently activates protein-kinase A (PKA) which augments the opening of sarcolemmal voltage-dependent calcium channel and sarcoplasmic ryanodine receptor Ca (2+) channels. The increased release of calcium increases myocardial contractility (Marx et al., 2000). Although cGMP is also increased in a small amount at low levels of NO, the upsurge of cAMP overrides the increase of cGMP and thus, the net effect of low NO is the increase of cardiac contractility (Rastaldo et al., 2007). At high concentration of NO, this activates guanylyl cyclase which catalyzes GTP to form cGMP (Tang et al., 2003). Larger amount of cGMP increases protein kinase G (PKG) which suppresses cardiac contractility (González et al., 2008). Shah et al. found that 8-bromo-cGMP reduced myocyte twitch amplitude and time to peak shortening. The negative inotropic effect of 8-bromo-cGMP on cardiomyocytes is due to the sensitivity decrease of the myofibrillar to calcium due to the activation of PKG (Shah et al., 1994).

NO has been shown to be overproduced in cirrhotic patients and experimental cirrhotic animals (Li et al., 1997; Huang et al., 2019). Therefore, NO plays a negative role in cardiac contraction in patients with cirrhosis. NO is thought to facilitate cGMP signaling. Using bile duct ligation (BDL) model, Van Obbergh et al. found that NO was overproduced in the development of cirrhotic cardiomyopathy. They showed that a nonselective NOS inhibitor, L-NMMA, restored the blunted contractile function of isolated heart from cirrhotic rats while it had no significant effect in control animals (Obbergh et al., 1996). Our group observed that iNOS is significantly increased in the heart of a cirrhotic rat (Liu et al., 2000). Furthermore, cGMP level is increased in cirrhotic ventricles compared with sham controls. The elevated serum and cardiac levels of cytokines like TNF-α suggest an underlying overactivation of cytokine/iNOS/cGMP pathway in cirrhosis (Liu et al., 2000). Facilitated cGMP further reduces calcium sensitivity of myofilaments (Shah et al., 1994) and inhibits β-adrenergic induced myocardial contraction (Takimoto et al., 2005).

### Carbon Monoxide

Carbon monoxide (CO) is the product of heme oxygenises (HO) in the body. There are two isoforms, one is inducible (HO-1; also known as heat shock protein 32), the other is constitutive (HO-2). These enzymes catalyze heme to biliverdin, ferrous ion, and CO. Similar to NO, CO triggers soluble guanylate cyclase to generate cGMP (Ewing et al., 1994). Raju et al. found that HO-1 mRNA was increased in the right ventricle in a canine model of congestive heart failure (Raju et al., 1999). Our lab identified HO-1 mRNA and protein expressions being increased in left ventricle of bile duct-ligated rats. We found that the overactivated HO-1 in cirrhotic heart was associated with an increase in left ventricular cGMP levels (Liu et al., 2001). Moreover, HO inhibitor, zinc protoporphyrin IX, reduced the elevated cGMP levels and restored the inhibited cardiac contractility in cirrhotic heart. These findings implicate the involvement of an HO-CO-cGMP pathway in the pathogenesis of cirrhotic cardiomyopathy.

### Other Substances

Galectin-3 is a beta-galactoside-binding lectin. Evidence of a pathogenic role for this compound derives from several observations. First, galectin-3 level is increased in serum from cirrhotic patients (Gudowska et al., 2015) and cirrhotic animal models (Yoon et al., Forthcoming 2022). Second, galectin-3 levels correlate with diastolic (Ansari et al., 2018) and systolic dysfunction (Zivlas et al., 2017). Third, following treatment of heart failure, galectin-3 levels were significantly decreased (Han et al., 2020). We found that galectin-3 is significantly increased in cirrhotic rat hearts, and a galectin inhibitor, N-acetyl-lactosamine, significantly improved cardiac function (Yoon et al., Forthcoming 2022). Another substance of interest is spermidine, a polyamine compound. In an acute myocardial infarction model in rats, Omar et al. (Omar et al., 2021) found that spermidine has cardiac protective effects. Sheibani et al. also demonstrated that spermidine has cardiac protective effects in a cirrhotic model in rats (Sheibani et al., 2020).

## Cardiac Receptors

### Beta-Adrenergic Receptor

It is well known that βAR plays a major role in cardiac contraction (Møller and Lee, 2018; Yoon et al., 2020). The activation of β-adrenergic receptors stimulates adenylyl cyclase generating cyclic AMP (cAMP) which activates protein kinase A, and ultimately phosphorylates cardiac contractile-related proteins, such as L-type calcium channels, phospholamban, troponin I, ryanodine receptors, and myosin-binding protein-C (Lee et al., 2007). Mashford et al. in the early 1960s found that cardiac responsiveness to exogenous infusions of catecholamines in patients with cirrhosis (Mashford et al., 1962) is attenuated. Our lab has found in animal models that the cardiac response to isoprenaline is significantly decreased in animal models of cirrhosis vs controls (Ma et al., 1996).

Specifically we found that compared with sham-operated controls, cirrhotic rats require a significantly higher dose of isoprenaline to raise basal heart rate by 50 beats/min (102 ± 19 vs. 28 ± 11 ng/kg). This was found to be in part due to a reduced myocardial βAR density and a higher dissociation constant. Subtype analysis demonstrated that it was the β1-AR subtype that accounted for the decrease of βAR density in cirrhotic hearts. However, the βAR affinity for agonist was not altered. We concluded based on this data that βAR downregulation was responsible for the myocardial hyporesponsiveness to catecholamines in the hearts of cirrhotic rats.

Although the mechanisms of the βAR density was not clearly elucidated, two hypotheses were described/proposed: 1) overdrive theory (Xiong et al., 2018), and 2) the presence of anti-βAR antibody (Ma et al., 2021). Xiong et al. demonstrated that the density of the cardiac βAR is decreased in patients with myocardial infarction whose circulating levels of catecholamines is increased. In cirrhotic patients, prolonged vasodilatation activates the sympathetic system which stimulates the β-adrenergic receptor leading to desensitization and dysfunction. Aberrations in the sympathetic nervous activity have been shown to be increased in cirrhotics (Henriksen et al., 1985). The chronic overdrive of sympathetic system gradually results in the reduction of βAR in heart.

Recently, our study found that the anti- β1-AR antibodies (anti- β1-AR) are increased in patients with CCM (Ma et al., 2021) compared with those without CCM. Patel et al. demonstrated that the anti-β1-AR binds to and constitutively stimulates the β1-AR to cause βAR desensitization and downregulation (Patel and Hernandez, 2013). We also found that anti-β1-AR is positively correlated to NT-proBNP, negatively correlated to left ventricular ejection fraction, fractional shortening, and the ratio of peak early (E wave) and atrial (A wave) flow velocities in CCM patients. Given this, Anti-β1-AR may prove to be a useful predictive biomarker for the presence of CCM. Since neutralization of Anti-β1-AR has therapeutic effects on dilated cardiomyopathy (Müller et al., 2000), we speculate that neutralization of Anti-β1-AR may also have therapeutic implications for CCM.

### M2-Muscarinic Receptor

Ventricular contractility is dependent on the interplay of stimulatory beta-adrenergic and inhibitory muscarinic receptors. Given this, the possible role of M2 receptors in CCM was investigated. Using a rat bile duct ligation model, our group found that the membrane M2 receptor density and binding affinity are similar between cirrhotic rats and controls. However, the magnitude of the negative inotropic response to carbachol was blunted in cirrhotic hearts. This suggests that the positive contractile response β-adrenergic and negative contractile response to M2-receptor are attenuated in cirrhotic hearts compared with controls (Jaue et al., 1997). A recent study demonstrated that both β1 and M2 receptor protein expressions are decreased in the myocardial tissues from cirrhotic rats induced by carbon tetrachloride (Yu et al., 2021). The difference might be due to the nature of liver injury in the animal models. Nonetheless, the abnormalities of cardiac contractile receptors suggest that cirrhosis is associated with a generalized defect in cardiac signaling pathways.

### Cannabinoid Receptors

Besides βAR and M2-receptor, cannabinoid receptors also play an important role in cardiac contractility. There are two CBRs, CB1R and CB2R. These two CBRs have different effects on end organ cardiac function. The activation of CB1 receptors is often in association with inflammation, cell death, reactive oxygen species (ROS) generation, inhibition of adenylyl cyclase *via* Gi/o-protein-dependent pathways, modulation of ion-channel function, and overall cardiac dysfunction (Rajesh et al., 2012; Moris et al., 2015). CB2 receptors in contrast play an important role in controlling tissue inflammation, inhibiting ROS, alleviating heart injury and overall exerts a beneficial effect on cardiomyocytes. (Moris et al., 2015; Pacher et al., 2018).

In cirrhotic cardiomyopathy, endocannabinoid plays an inhibitory role in cardiac contraction (Gaskari et al., 2015). Our lab revealed that endocannabinoids are increased locally in the hearts of cirrhotic rats. We observed a dose-response curve of papillary muscle from cirrhotic rates toa beta-adrenergic agonist isoproterenol being significantly blunted. Administration ofCB-1 antagonist AM251 completely restored this dose-response curve. Force-frequency relationship studies found that at higher frequencies, anandamide reuptake blockers (VDM11 and AM404) also significantly enhanced muscle relaxation in cardiomyocytes from cirrhotic rats, but not in controls. This effect is entirely blocked by AM251. These findings suggests the pathogenic role of endocannabinoids in cirrhotic cardiomyopathy.

### Abnormalities of Ion Kinetics

Cardiomyocyte function depends on coordinated movements of calcium into and out of the cell. Calcium combines with myofilaments and plays the central role in cardiomyocyte contraction. Calcium enters the myocyte through plasma membrane calcium channels and is stored in the sarcoplasmic reticulum. Stimulation of the sarcoplasmic reticulum by ryanodine or caffeine releases the calcium). Unlike skeletal muscle, cardiac contractile initiation in cardiomyocyte is totally dependent on the extracellular calcium. The extracellular calcium influx to the cytoplasm via L-type calcium channels and trigger calcium release from sarcoplasmic reticulum, which then participates in the cardiomyocyte contraction. It has been characterized that the dysregulation of myocardial calcium is a hallmark of chronic heart failure 29,621,141 which may also be playing a role in cirrhotic cardiomyopathy.

We investigated the status of the cellular calcium-regulatory system in the cirrhotic rat and found that membrane bound L-type calcium channel, a calcium influx regulator, is significantly decreased compared with that in control myocytes (Ward et al., 2001). There were no significant difference in the transcripts or protein involved in the intracellular calcium-handling system, the sarcoplasmic reticulum and RyR between cirrhotic and control hearts (Ward et al., 2001). Our data indicated that the plasma membrane calcium channels are quantitatively reduced and functionally depressed, whereas intracellular signalling components are intact.

Lastly potassium is another ion that is involved in cirrhotic cardiomyopathy. Our lab explored the underlying mechanisms for the electrophysiological abnormalities in cirrhotic model in rats and found that BothCa2+ -independent transient outward and K+ current delayed rectifier K+ current were significantly decreased which contribute to the prolonged Q-T interval that can seen from electrocardiograms of cirrhotic patients (Ward et al., 1997).

### Potential Therapeutic Targets of Mechanistic Pathways

Liver transplantation is the definitive ‘cure’ for CCM. However, as it is expensive, technically and logistically complex, and not routinely available in all global regions, a medical therapy for CCM is highly desirable. Unfortunately, at present, such medical treatment is still an unmet need. Based on many of the aforementioned mechanisms, several attractive potential targets for therapeutic intervention may be suggested. An obvious one is the beta-adrenergic system, as beta-blockers have been demonstrated to help protect the heart in some noncirrhotic types of heart failure or dysfunction, for example in hypertrophic cardiomyopathy or catecholamine cardiotoxicity. Furthermore, nonspecific beta-adrenergic blockers (NSBBs) attenuate portal pressure, improve mesenteric venous congestion and intestinal permeability and therefore indirectly alleviate systemic inflammation. Moreover, beta-adrenergic blockers directly and indirectly reduce serum TNFα and interleukin-6 and help preserve intestinal barrier function in patients with sepsis, shock, and other critical illnesses (Tan et al., 2021). The reduction of systemic inflammation also decreases inflammatory cell infiltration in the cirrhotic heart. Another potential favourable effect of NSBBS is correcting the prolonged QTc interval and thus decreasing the risk of ventricular arrhythmias (Yoon et al., 2021). However, a randomized-controlled trial showed that 6 months of treatment with β-blocker had no effect on CCM either in function or morphology (Silvestre et al., 2018).

The sulfonic acid taurine, a major component of bile, has pleiotropic protective properties on heart diseases (Bailout et al., 2021). It has anti-oxidative, anti-inflammatory and anti-apoptotic effects (Ma et al., 2022). Furthermore, taurine decreases bile acid concentrations. Liu and colleagues (Liu et al., 2020) reported that taurine has a protective effect on transverse aortic constriction-induced heart failure. The protective effects of taurine on heart failure may also apply to CCM, but need further study.

Spermidine has pleiotropic functions such as anti-oxidation and anti-inflammation. Sheibani et al. (2020) found that spermidine increases superoxide dismutase, an antioxidative enzyme and decreases malondialdehyde, a marker for oxidative stress. Furthermore, spermidine decreases TNFα and IL-β. It improves the contractility in isolated papillary muscle from cirrhotic heart. Spermidine therefore is a potential agent for the treatment of CCM.

TNFα is also a potential therapeutic target for CCM (Yang et al., 2010). Circulating levels of TNFα correlate with the severity of heart failure (Kosar et al., 2006). Our study in mice showed that plasma TNFα was increased, and cardiac contractility was blunted in BDL mice. Anti-TNFα treatment in BDL mice led to the correction of cardiomyocyte contractile dysfunction, decreased cardiac anandamide, nitric oxide, oxidative stress and NFκB/These last four factors are well known cardiac contractile inhibitors. Inhibiting or abrogating TNFα may thus be a potentially useful therapeutic strategy to restore cardiac contractility in cirrhotic patients.

There are complex inter-relationships between these pathways. β-blockers, taurine and spermidine all have anti-apoptotic, anti-oxidative and anti-inflammatory effects (Sheibani et al., 2020; Liu et al., 2021; Tan et al., 2021). Moreover, although CCM is etiology-independent, it is possible that the specific pathogenic mechanisms of cardiac dysfunction in patients or animal models of cirrhosis may differ according the cause of cirrhosis, eg, alcohol-associated vs autoimmune or viral. Whether “precision medicine” is also applicable to cirrhotic cardiomyopathy needs further investigation.

**FIGURE 2:**
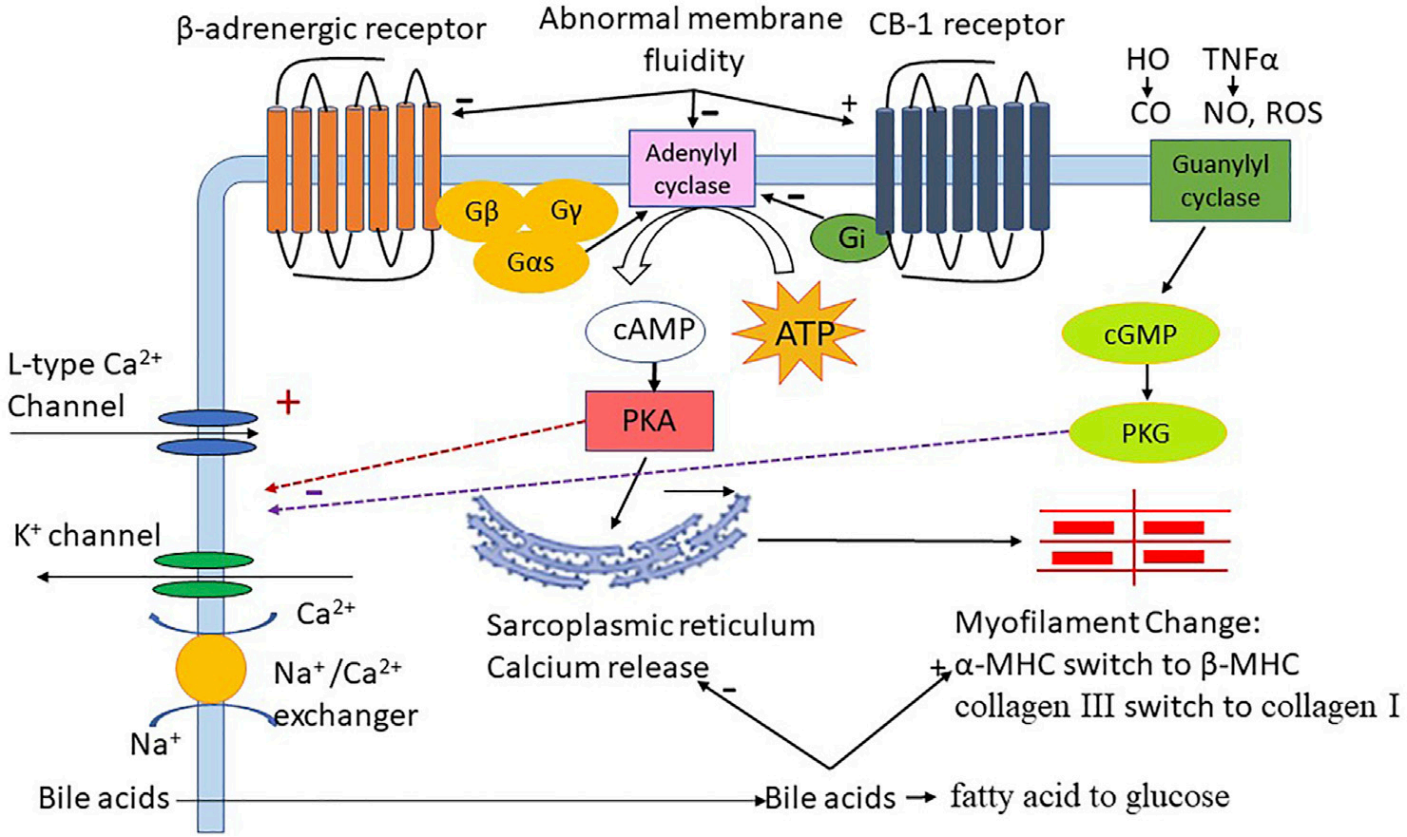
The role of different factors in cirrhotic cardiomyopathy; HO:heme oxygenase; CO:carbon monoxide, TNFα:tumor necrosis factor-alpha; NO:nitric oxide, ROS: reactive oxygen species, Gβ Gγ Gas Gi G-protein subunit; ATP: adenosine triphosphate, cAMP: 3′,5′-cyclic adenosine monophosphate; cGMP: 3′,5′-cyclic guanosine monophosphate, PKA: protein kinase A, PKG: protein kinase G; FAO: fatty acid oxidation; MHC: Myosin heavy chain.

In conclusion, perturbations within the luminal environment due to cirrhosis and the circulatory changes associated with portal hypertension can lead to adverse cardiac outcomes. The luminal variables upstream from cirrhosis cardiomyopathy are still yet to be defined in the patient setting. Both local immune and parenchymal changes within the heart occurring as a result of the systemic stress of cirrhosis can contribute to cardiac dysfunction. The pathogenic mechanisms underlying cirrhotic cardiomyopathy are complex and affected by many different parameters (Figure 2). The factors that affect cardiac function in common heart diseases also impact cardiac function in cirrhotic cardiomyopathy. Based on the pathogenic mechanisms, we suggest randomized clinical trials in the future studies to test the therapeutic effect, including neutralization of bile acids, anti-oxidants, anti-inflammatory agents and methods to improve liver function.
